# Causal Relationship between Celiac Stenosis and Pancreaticoduodenal Artery Aneurysm: Interpretation by Simulation Using an Electric Circuit

**DOI:** 10.1155/2020/2738726

**Published:** 2020-06-08

**Authors:** Hye Jeong Yoon, Jeong Sik Choi, Woo Young Shin, Keon-Young Lee, Seung-Ik Ahn

**Affiliations:** Department of Surgery, Inha University School of Medicine, Incheon 22332, Republic of Korea

## Abstract

Pancreaticoduodenal artery (PDA) aneurysm and celiac artery (CA) stenosis are rare diseases in themselves. Interestingly, however, there are more cases documented in the literature in which these two disease entities occurred together than could be coincidental, and CA stenosis has been suggested as the provocative condition in developing PDA aneurysm. This study is aimed at examining the causal relationship between CA stenosis and PDA aneurysm by simulating the splanchnic circulation with an electric circuit. A patient with multiple PDA aneurysms and collaterals with CA stenosis was treated in our institution using hybrid techniques. The patient's pre- and postoperative status was simulated using an electric circuit, and the two possible scenarios were tested for compatibility: the stenosis-first scenario *vs.* the aneurysm-first scenario. The simulation was performed in two ways: using Simulink® software (MATLAB® Release 2018b) and actual circuit construction on a breadboard. The stenosis-first scenario showed that as the CA stenosis progresses, the blood flow through PDA increases, favoring the development of an aneurysm and/or collaterals if the artery was already compromised by a weakening condition. On the other hand, the aneurysm-first scenario also showed that if the aneurysm or collaterals developed first, the aneurysm will steal the blood flow through the CA, causing it to collapse if the artery was already compromised by increased wall tension. Contrary to the common belief, this study showed that in patients suffering from concurrent CA stenosis and PDA aneurysm, either condition could develop first and predispose the development of the other. The simulation of splanchnic blood flow with an electric circuit provides a useful tool for analyzing rare vascular diseases that are difficult to provoke in clinical and animal studies.

## 1. Introduction

Visceral artery aneurysm (VAA) is a rare but potentially lethal disease with diverse causes [1–3]. Aneurysms of pancreaticoduodenal arcades are rarer and comprise about 2% of all VAAs [4]. Interestingly, pancreaticoduodenal artery (PDA) aneurysms are commonly accompanied by celiac artery (CA) stenosis or occlusion, which is also rare [5, 6]. It is unclear whether the relationship between VAA aneurysm and aortic take-off vascular stenosis is causal or coincidental [7]. It is generally believed that the increase in the collateral flow due to stenosis or occlusion of the major aortic branches causes aneurysmal dilatation of the representative arteries [5, 7, 8]. However, because of the rarity of both disease entities, it is difficult to collect sufficient evidence to support this theory through prospective studies or animal provocation experiments. In a previous study, we proposed an electric simulation model for portosystemic shunt and showed that the splanchnic circulation can be simulated electrically because the electric current and blood flow should follow the Ohm's law [9]. In this article, we will focus on the arterial side of the intra-abdominal blood flow and develop another electric model with two adjustable points that represent CA stenosis and PDA aneurysm. The electric simulation model was run by simulation software and actual electric circuit construction on a breadboard. Using this simulation, we show which event came first, the stenosis or the aneurysm. The clinical counterpart would be our previous case, in which we observed reconstitution of the collapsed CA after the ablation of PDA aneurysms and collaterals without recanalization of the CA stenosis [10].

## 2. Materials and Methods

### 2.1. Case Presentation [10]

**Figure 1:**
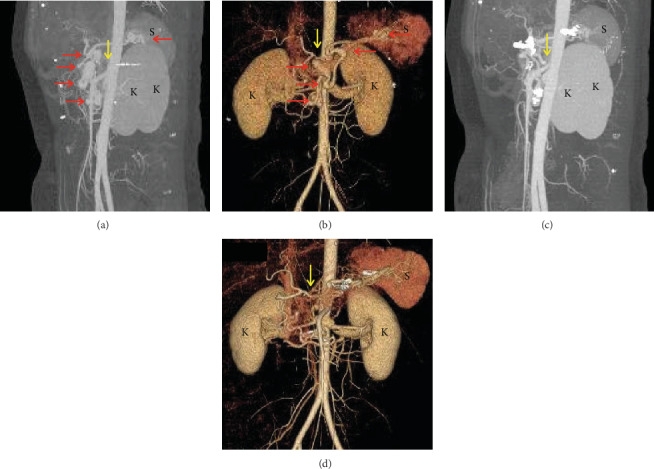
Three-dimensional angiomesenteric computed tomogram of a 37-year-old woman with multiple visceral artery aneurysms (VAAs). (a, b) Preoperative scan showing saccular or fusiform VAAs in the pancreaticoduodenal, dorsal pancreatic, and transverse pancreatic arteries (red arrows). Celiac artery (CA) stenosis with collapsed common hepatic and proximal splenic arteries is visible (yellow arrows). (c, d) On a follow-up scan 66 months after VAA resection and clipping, no recurrence is identified. Note that the collapsed CA branches were reconstituted without the relief of CA stenosis (yellow arrows). K: kidneys; S: spleen.

A 37-year-old multiparous woman was referred to our hospital for asymptomatic multiple VAAs on her pancreaticoduodenal arcades. Three-dimensional angiomesenteric computed tomography (3D-AMCT) showed five fusiform and saccular aneurysms in her inferior PDA, dorsal pancreatic, and transverse pancreatic arteries (Figures 1(a) and 1(b)). The aneurysms were managed by open surgical resection and anastomosis and clipping. The CA stenosis was left in situ. Pathologic examination of the resected aneurysm showed fibrosis and myxoid degeneration of the arterial wall. Follow-up 3D-AMCT was performed at 6-month to 1-year intervals until 66 months postoperative but showed no evidence of aneurysm recurrence with unchanged CA stenosis. Notably, the collapsed common hepatic and splenic arterial take-off segments were reconstituted from the immediate postoperative follow-up (Figures 1(c) and 1(d)).

### 2.2. Electric Circuit Model of the Splanchnic Circulation

**Figure 2:**
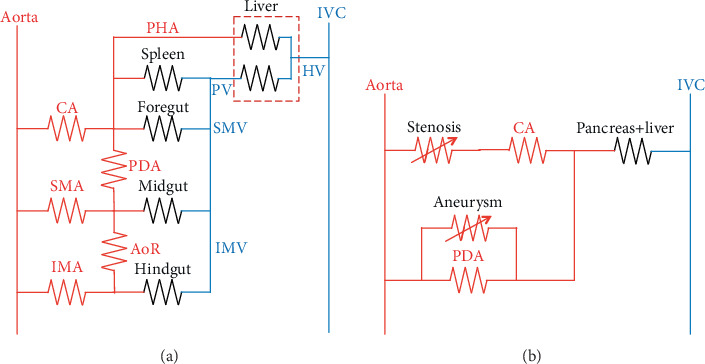
Schematic diagram of splanchnic vascular connections using electric circuit symbols. (a) The spatial relations and differences in resistance are ignored. (b) The final basic configuration of connections was simplified using the circuit theory. Note that the stenosis and the aneurysm are represented by potentiometers. CA: celiac artery; SMA: superior mesenteric artery; IMA: inferior mesenteric artery; PHA: proper hepatic artery; PDA: pancreaticoduodenal artery; AoR: arc of Riolan; IVC: inferior vena cava; HV: hepatic vein; PV: portal vein; SMV: superior mesenteric vein; IMV: inferior mesenteric vein.

The schematic diagram of splanchnic vascular connections is presented in Figure 2(a). For easy viewing, spatial relations of internal organs and relative differences in resistance were ignored. At a glance, it is evident that all intra-abdominal circulations converge into the portal vein, except the proper hepatic artery, which in turn converges with the hepatic vein in the liver. Any extra connection between the portal vein and inferior vena cava would form a portosystemic shunt, which is normally just a potential connection with high resistance and was omitted from the diagram. Using Ohm's law and circuit theory, any number of resistances can be replaced with a single equivalent resistance irrespective of the connection mode (series or parallel). The electric circuit model in Figure 2(a) can be simplified into the basic configuration shown in Figure 2(b), leaving only the essential elements of interest. The vascular stenosis can be simulated by a potentiometer connected in series, while an aneurysm or collaterals can be simulated by a potentiometer connected in parallel.

### 2.3. Simulation of PDA Aneurysm with CA Stenosis Using an Electric Circuit

The conversion of blood flow to an electric current was arbitrarily done using the following conversion factors:

1. Pressure: 1 mmHg = 0.1 V (e.g., 120 mmHg = 12 V).
2. Flow rate: 1 L/min = 1 mA (e.g., portal flow [11] 0.86 L/min = 0.86 mA).
3. Resistance: pressure/flow rate (e.g., portal venous resistance = 10 mmHg [12]/0.86 L/min = 1 V/0.86 mA = 1163 *Ω* ≒ 1.2 k*Ω*).

### 2.4. Construction of Electric Simulation Circuit Using Software

**Figure 3:**
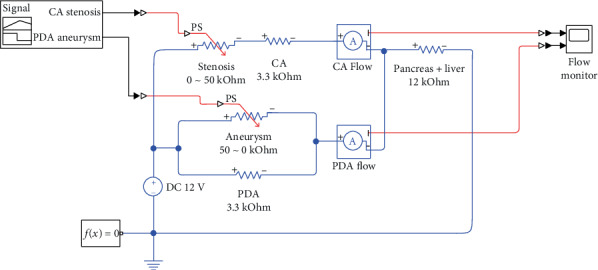
Construction of electric simulation circuit using Simulink® software. A signal generator controls CA stenosis and PDA aneurysm resistances at a given time. The resulting CA and PDA flows are presented on the flow monitor. CA: celiac artery; PDA: pancreaticoduodenal artery; DC: direct current; PS: potentiometer signal; *f*(*x*) = 0: Simulink function block.

The representative circuit in Figure 2(b) was constructed using Simulink® (MATLAB® Release 2018b; The Mathworks Inc., Natick, MA, USA) (Figure 3). The resistance of each resistor was calculated as follows:

1. Resistance of CA and PDA: 1.3 k*Ω* [13] × 2 = 2.6 k*Ω* ≒ 3.3 k*Ω* (available resistor).
2. Voltage drop across CA and PDA: (3.3 k*Ω*/2) × 0.86 mA = 1.4 V.
3. Resistance of pancreas+liver: (12 V – 1.4 V)/0.86 mA = 12.3 *Ω* ≒ 12 k*Ω* (available resistor).

The CA stenosis and PDA aneurysm were simulated using potentiometers ranging from 0 to 50 k*Ω*, respectively (available potentiometer). The resistance of the potentiometers was determined by signal generators. The direct current source was set to 12 V. The CA and PDA flows were detected using current sensors, and the tracings were recorded using flow monitors. We had tested two scenarios, and any manipulation in each scenario was set to 1 second. In scenario 1, we assumed that the CA stenosis came first (stenosis-first scenario) and increased the resistance of the CA potentiometer from 0 *Ω* to 50 k*Ω*, while the PDA potentiometer was set to 50 k*Ω*. After the CA potentiometer reached 50 k*Ω*, the resistance of the PDA potentiometer was decreased from 50 k*Ω* to 0 *Ω* to simulate the formation of an aneurysm and collaterals. The PDA potentiometer was increased back to 50 k*Ω*, simulating the postoperative status. The CA and PDA flow tracings were recorded along with the entire scenario. In scenario 2, we assumed that the PDA aneurysm came first (aneurysm-first scenario) and decreased the resistance of the PDA potentiometer from 50 k*Ω* to 0 k*Ω*, while CA potentiometer was set to 0 *Ω*. After the PDA potentiometer reached 0 *Ω*, the CA potentiometer was increased from 0 *Ω* to 50 k*Ω* to simulate the development of stenosis. While the CA potentiometer was set to 50 k*Ω*, the resistance of the PDA potentiometer was increased back to 50 k*Ω* to simulate the postoperative status. The CA and PDA flow tracings were recorded with the entire scenario. The observations were focused on changes in the CA and PDA flow and whether the CA flow can be reconstituted after the correction of the PDA aneurysm.

### 2.5. Construction of Actual Electric Circuit

**Figure 4:**
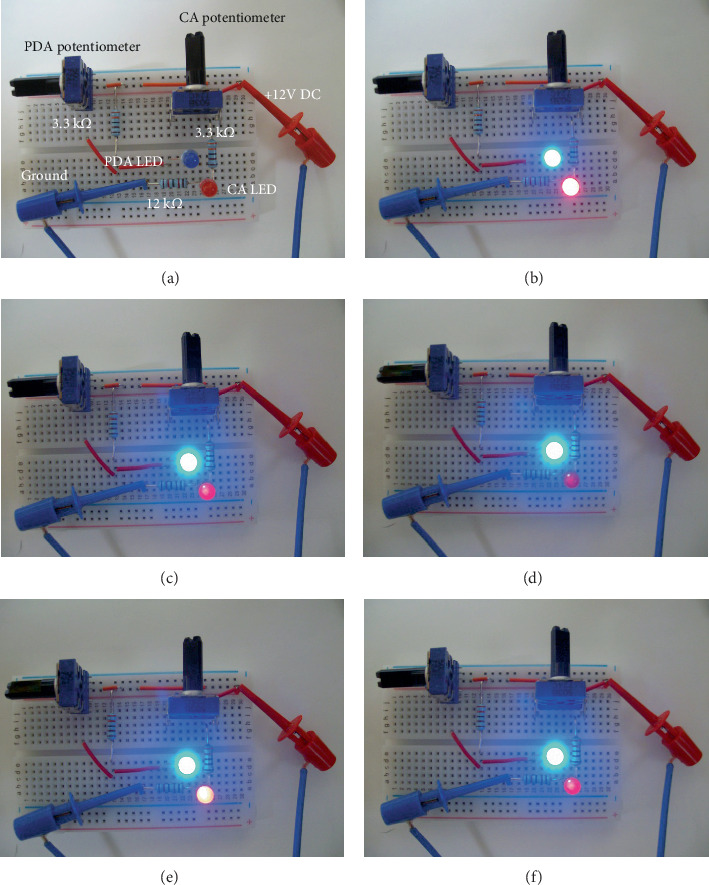
Actual construction of an electric simulation circuit on a breadboard. Note that the dimension of the breadboard is 8.2 cm × 5.4 cm. (a) No voltage source applied. (b) 12 V direct current (DC) applied, with the CA potentiometer set to 0 *Ω* and the PDA potentiometer to 50 k*Ω*, respectively, simulating normal status. (c) The CA potentiometer was set to 50 k*Ω*, while the PDA potentiometer remained at 50 k*Ω*, simulating the CA stenosis without the PDA aneurysm. Note that the PDA light-emitting diode (LED) became brighter than the normal status, suggesting an increase in PDA flow. (d) The CA potentiometer remained at 50 k*Ω*, and the PDA potentiometer was decreased to 0 *Ω*, simulating CA stenosis with PDA aneurysm, the preoperative status of the presented case. Note that the CA LED is almost off, suggesting the absence of the CA flow. (e) The CA potentiometer was set to 0 *Ω*, while the PDA potentiometer was set to 0 *Ω*, simulating PDA aneurysm without CA stenosis. The PDA LED became brighter than the normal status without dimming of the CA LED. (f) The CA potentiometer was set to 50 k*Ω*, and the PDA potentiometer was set to 50 k*Ω*, simulating the postoperative status of the presented case. Note that the CA LED was dimly lit, suggesting reconstitution of the CA flow. CA: celiac artery; PDA: pancreaticoduodenal artery.

An actual electric circuit was constructed using electronic parts on a breadboard based on the diagram shown in Figure 2(b) (Figure 4(a)). To inspect electric current visually, the CA and PDA flows were detected using red and blue light-emitting diodes (LEDs), respectively. The CA potentiometer was initially set to 0 *Ω* and the PDA potentiometer to 50 k*Ω* to simulate normal status. In the stenosis-first scenario, the CA potentiometer was increased in resistance to model the development of stenosis, while the PDA potentiometer was set to 50 k*Ω*. The brightness of the PDA LED was examined if it was increased along with the increase in the CA potentiometer. Once the CA potentiometer had increased to 50 k*Ω*, the PDA potentiometer was decreased in resistance to model the development of an aneurysm. In the meantime, the brightness of the CA LED was examined if it was decreased to simulate the CA collapse. Finally, the PDA potentiometer was increased to simulate the postoperative condition. The CA LED was examined if it was dimly lit to represent the clinical reconstitution of CA flow. In the aneurysm-first scenario, the sequence of CA and PDA potentiometer manipulations was reversed, and changes in the brightness of both LEDs were observed if they could result in the same condition initially and postoperatively as the stenosis-first scenario.

## 3. Results

### 3.1. Electric Simulation Using Software

**Figure 5:**
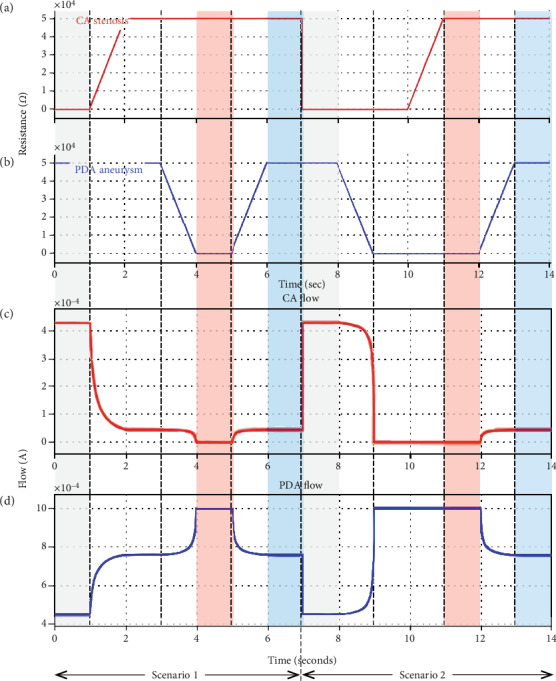
Tracings of the signal generator and the flow monitor of the Simulink® electric simulation model arranged according to the timeline. (a, b) The resistance of the CA potentiometer and the PDA potentiometer, respectively. (c, d) The CA flow and the PDA flow, respectively. The same colored zones represent the same clinical status in each scenario. Note that the same clinical results can be reached through both scenarios. CA: celiac artery; PDA: pancreaticoduodenal artery.

Changes in the resistance of CA stenosis and PDA aneurysm (Figure 5(a) and (b), respectively) and corresponding changes in CA and PDA flow according to time (Figure 5(c) and (d), respectively) are presented in Figure 5. In scenario 1 (from 0 to 7 second), the resistances of CA stenosis and PDA aneurysm were initially set to 0 *Ω* and 50 k*Ω*, respectively, to simulate normal status (from 0 to 1 sec). The CA flow was 0.427 mA (corresponding to a blood flow of 427 mL/min), and the PDA flow was 0.455 mA (first light-grey zone in Figure 5). From 1 to 2 sec, the resistance of CA stenosis increased from 0 *Ω* to 50 k*Ω* to simulate the development of stenosis, while that of PDA aneurysm remained at 50 k*Ω* (without an aneurysm). The CA flow decreased from 0.427 mA to 0.044 mA (44 mL/min), whereas the PDA flow increased from 0.455 mA (blood flow, 455 mL/min) to 0.760 mA (blood flow, 760 mL/min). From 2 to 3 sec, CA stenosis without the development of PDA aneurysm was simulated. The development of PDA aneurysm was simulated from 3 to 4 sec. The resistance of PDA aneurysm decreased from 50 k*Ω* to 0 *Ω*, while that of CA stenosis remained at 50 k*Ω*. The CA flow decreased from 0.044 mA to 0.000 mA (0 mL/min), whereas the PDA flow increased 0.760 mA to 1.000 mA (1,000 mL/min). From 4 to 5 sec, severe CA stenosis with full-blown PDA aneurysm and collaterals is simulated to show the patient at initial presentation. The CA flow was 0 mL/min, and the PDA flow was 1,000 mL/min, meaning a total shunt of blood flow through PDA (first light-red zone in Figure 5). From 5 to 6 sec, the correction of PDA aneurysm and collaterals is simulated. The resistance of PDA aneurysm increased from 0 *Ω* to 50 k*Ω*, while that of CA stenosis remained at 50 k*Ω*. The CA flow increased from 0.000 mA to 0.044 mA, whereas the PDA flow decreased from 1.000 mA to 0.760 mA. From 6 to 7 sec, postoperative status is simulated. Note that the CA and PDA flow never resumed normal status because the CA stenosis was left uncorrected. Also, the CA flow was 44 mL/min and not 0 mL/min, representing the reconstitution of CA as in the presented patient (first light-blue zone in Figure 5). From 7 to 14 sec, scenario 2 was shown. Again, normal status was represented from 7 to 8 sec, with the resistance of CA stenosis and PDA aneurysm set to 0 *Ω* and 50 k*Ω*, respectively (second light-grey zone in Figure 5). From 8 to 9 sec, the resistance of PDA aneurysm decreased from 50 k*Ω* to 0 *Ω* to simulate the development of an aneurysm, while that of CA stenosis remained at 0 *Ω*. The PDA flow increased from 0.455 mA (455 mL/min) to 1.000 mA (1,000 mL/min), whereas the CA flow decreased from 0.427 mA (427 mL/min) to 0.000 mA (0 mL/min), representing a total shunt of blood flow through the PDA aneurysm. From 9 to 10 sec, a PDA aneurysm without CA stenosis is simulated. The development of CA stenosis was simulated from 10 to 11 sec, in which the resistance of CA stenosis increased from 0 *Ω* to 50 k*Ω* and that of PDA aneurysm remained at 0 *Ω*. Neither CA nor PDA flow had changed; rather, they remained at 0.000 mA and 1.000 mA, respectively. From 11 to 12 sec, severe CA stenosis with full-blown PDA aneurysm was simulated to show the patient at the initial presentation (second light-red zone). From 12 to 13 sec, correction of the PDA aneurysm was simulated. The resistance of PDA aneurysm increased from 0 *Ω* to 50 k*Ω*, while that of CA stenosis remained at 50 k*Ω*. The CA flow increased from 0.000 mA to 0.044 mA, whereas the PDA flow decreased from 1.000 mA to 0.760 mA (760 mL/min). From 13 to 14 sec, postoperative status was simulated (second light-blue zone in Figure 5). Note that the first and second color-augmented zones in the timeline of Figure 5 indicate the same values, suggesting that the clinical settings of normal, preoperative, and postoperative conditions can be reached through either scenario.

### 3.2. Actual Electric Simulation Model Constructed on a Breadboard

In Figure 4(a), no voltage source was connected, and the CA LED and PDA LED were off. In Figure 4(b), DC 12 V were supplied with the CA potentiometer set to its minimum resistance (0 *Ω*) and PDA potentiometer to its maximum (50 k*Ω*) to simulate normal status. The CA and PDA LEDs were on, indicating normal CA and PDA blood flow.  Figure 4(c) simulates scenario 1. With the PDA potentiometer set to 50 k*Ω*, the CA potentiometer was increased in resistance, simulating CA stenosis. When the CA potentiometer reached 50 k*Ω*, the CA LED was dimly lit and the PDA LED was brighter, indicating increased blood flow through the PDA. In this setting, the PDA potentiometer decreased in resistance to simulate the development of aneurysm. When the PDA potentiometer reached 0 *Ω*, the CA LED was almost off, indicating practically no blood flow through CA (Figure 4(d)). The PDA potentiometer increased in resistance again to simulate the postoperative state. Naturally, the CA and PDA LEDs went back to the initial status as in Figure 4(c) (Figure 4(f)). Notably, the CA LED lit brighter than that of Figure 4(d), meaning that the collapsed CA had been clinically reconstituted.  Figure 4(e) simulates scenario 2. With the CA potentiometer set to 0 *Ω*, the PDA potentiometer was decreased in resistance, simulating aneurysm formation. When the PDA potentiometer reached 0 *Ω*, the CA LED became dimmer and PDA LED became brighter than those in Figure 4(b), showing that the CA blood flow can be decreased without the development of CA stenosis. In this setting, the CA potentiometer was increased in resistance to simulate the development of CA stenosis. With the CA potentiometer reaching 50 k*Ω*, the CA LED turned off as shown in Figure 4(d). The PDA potentiometer increased in resistance to simulate the postoperative state. Again, the CA and PDA LEDs achieved status as shown in Figure 4(f). The sequence of events in scenario 1 was (b)–(c)–(d)–(f), while that in scenario 2 was (b)–(e)–(d)–(f) of Figure 4, respectively, showing that the same preoperative and postoperative clinical conditions could be reached through either scenario.

## 4. Discussion

Both being rare disease entities, the high rate of up to 80% of concurrent CA stenosis and PDA aneurysm suggested a causal rather than coincidental relationship between the two conditions [14, 15]. It is generally believed that the increase in the collateral flow due to stenosis or occlusion of the major aortic branches caused aneurysmal dilatation of the representative arteries [5, 7, 8, 16]. However, it is possible that aneurysmal dilatation of the collateral arteries developed first, shunting the blood flow away from the major visceral arteries and causing it to collapse if their take-off point from the aorta was already tensed, as in median arcuate ligament syndrome [6]. The causal relationship is important for deciding whether to treat stenotic vessels and evaluating aneurysm recurrence [14, 16]. For arterial stenosis to be further confirmed as the cause or precondition of the development of an aneurysm, stenosis must precede the aneurysm, not vice versa. Considering the rarity of both diseases, however, it would be difficult, if not impossible, to observe the sequence of events by clinical study or animal experiments.

Blood circulation can be simulated by an electric circuit because both are flow systems with different substances and scales and expected to obey the Ohm's law and, hence, circuit theory [13]. Many studies performed so far had adopted electronic simulation methodology in the study of the cardiovascular system, and most of them were quantitative analyses using numerical modeling [13, 17, 18]. In contrast, our current study was a qualitative one, focused only on the causal relationship between CA stenosis and PDA aneurysm and not on the actual numerical values. In a previous study, we simulated hepatic portal circulation using an electric circuit [9]. In this study, we applied the same principle to the preportal abdominal vasculature to simulate the arterial system. As for the clinical case to model, we adopted the patient we reported previously [10]. The patient had multiple VAAs in the PDA, splenic, and peripancreatic arteries with CA stenosis at its origin. The case was peculiar in that it had reported the postoperative reconstitution of previously collapsed CA distal to the stenotic point. Chiou et al. also reported a case of PDA aneurysm with CA occlusion and restoration of the gastroduodenal and splenic arterial pulses after resection anastomosis of the aneurysm without CA recanalization [3]. This clinically insignificant finding is of utmost importance in the pathophysiological point of view since it can verify that the electric simulation model is accurately representing the real case. A collapsed artery means 0 pressure gradients, that is, the same pressure between the origin and the insertion of the artery. Clinically, it means abundant collaterals with a total steal phenomenon. The finding that it is reconstituted after surgery represents there should be an increase in resistance in the collateral systems, resulting in the centrifugal blood flow; if the flow existed, it cannot be in the reverse direction because pressure in the arterial system cannot be higher than that in the aorta. In the simulation model, this basic characteristic is represented by an LED that is on when the electric current flows in one direction only.

This study consisted of two parts with two scenarios each. In the first part, the electric circuit simulating the simplified abdominal arterial circulation was constructed using Simulink® software. It alleviated the actual challenge to build a real circuit, thereby saving time and effort required to prepare the required electronic parts and measuring equipment. At the same time, the software enabled us to measure the exact values of resistance and electric current at a given time and returned the results in clear digital charts. It embedded self-testing function, which alarmed if the circuit had structural errors. However, being a virtual electric circuit, it was difficult to perceive instinctively because we cannot see or feel what is happening inside. In the second part of this study, we realized the simulation circuit on a breadboard. Using LEDs, we tried to represent blood flow with the light of an LED. Although it cannot be measured exactly, the amount of blood flow can be perceived by the LED brightness. The two scenarios represent the chronological sequence of events, that is, whether the development of CA stenosis or PDA aneurysm occurred first. Each scenario begins with a normal status; the absence of neither the stenosis nor the aneurysm, goes through the existence of both conditions, and ends with the postoperative status in which CA flow had been reconstituted with the stenosis in situ. We focused on the effect of one condition on the other, if CA stenosis increases the likelihood of developing PDA aneurysm or vice versa.

Some basic considerations are required. For blood vessels, the resistance is proportional to the length and inversely proportional to the 4^th^ power of the radius of the vessel, whereas in electric circuit, the resistance is proportional to the length and inversely proportional to the square of the radius of the wire. A stenotic or occluded vessel increases the vascular resistance, an increase in electric resistance in the simulation model. It can be simulated with resistors connected in series or an increase in the resistance of a potentiometer. If a potentiometer is added in series to a fixed resistor, the combined resistance cannot be less than the latter. On the other hand, an aneurysm, whether saccular or fusiform, indicates an increase in the vascular diameter and a decrease in the flow resistance [17, 18]. The occurrence of turbulent flow in an aneurysm can act as a resistance to the flow. However, Hassani et al. observed that the pressure drop across an aneurysm decreased as the diameter of the aneurysm increased from 20% to 90% of its original diameter, irrespective of the aneurysm topology [17]. Their findings implicate that the resistance to blood flow decreases as the diameter of an aneurysm increases, and in practical purposes, turbulence is not a significant factor. Although they adopted a computational fluid dynamics method in their analysis, it is reported to agree well with the realistic measurements [19]. From the fluid dynamics point of view, an aneurysm is like the development of collateral circulations in terms of cause and effect; actually, they commonly occur together [5, 8]. In the electric model, an aneurysm or collateral vessels can be simulated with resistors connected in parallel or a decrease in the resistance of a potentiometer. If a potentiometer is added in parallel to a fixed resistor, the combined resistance cannot be greater than the latter. Another thing to consider is as follows: by LaPlace's law, vascular wall tension (T) is proportional to the radius (*R*) of the vessel for a given intravascular pressure (*P*) (*T* = *PR*). An increase in the internal pressure predisposes the development of an aneurysm by increasing the vascular wall tension for a given radius. This has been the proposed mechanism underlying the pathogenesis of PDA aneurysm in the presence of CA stenosis [5]. However, LaPlace's law defines correlations between pressure, radius, and wall tension but not their sequence of events; that is, a decrease in the internal pressure could predispose the development of stenosis by decreasing wall tension for a given radius. In short, stenosis and an aneurysm are a different manifestation of one continuum; either can cause the other.

Figure 2(a) shows the electric model of the splanchnic circulation. Using the lumped element model and circuit theory, it can be stepwise simplified as shown in Figure 2(b), leaving only the components of interest. Each parameter was determined by converting the reported portal flow [11] to an electric current with readily available electric components and a safe-to-manipulate scale. The basic configuration of the CA and PDA is resistors connected in parallel, a current divider. The sum of the flow through the CA and PDA is the portal flow. The decrease in the CA flow due to stenosis increases the PDA flow and vice versa. In this configuration, any sequence of events is possible, either the CA stenosis-first scenario (scenario 1) or the PDA aneurysm-first scenario (scenario 2). Figure 5 shows the results of the first part of this study. Note that the tracings in Figure 5(c) and (d) are mirror images about the time axis that reflect the current divider configuration of the CA and PDA. The results showed that the preoperative condition (time interval 4–5 sec and 11–12 sec) can be reached with either scenario, with the same clinical postoperative status (time interval 6–7 sec and 13–14 sec). Miyahara et al. [20] developed an electric model of PDA aneurysm with CA stenosis and showed that as the CA stenosis progressed, the flow through the anterior and posterior PDA decreased, changed their direction, and then increased drastically. They concluded that this increase in the PDA flow might have contributed to the development of a PDA aneurysm. Their study was comparable to scenario 1 in our study. However, they did not test whether the increase in PDA flow could decrease the CA flow, predisposing the individual to the development of a CA stenosis (scenario 2). Moreover, they set the aortic pressure to 13 kV, an immediately lethal voltage when contacted and difficult to realize practically. The second part of our study, on the contrary, consisted of safe voltage and electric components that are readily available for a person with an interest in electronics and could be repeated manually as many times as to obtain intuitive insight. Like in the virtual simulation model, any sequence of events could result in the pre- and postoperative status compatible with our case. It demonstrated that CA stenosis and PDA aneurysm (and/or collateral vessels) are required for the CA to collapse as in the presented case unless it is completely occluded.

This study showed that CA stenosis and PDA aneurysm can provoke each other in reverse directions. CA stenosis provokes PDA aneurysm by increasing PDA flow if it is already compromised with wall tension-weakening conditions such as segmental arterial mediolysis, Marfan syndrome, or atherosclerosis [3, 19]. Conversely, PDA aneurysm and/or arterial collaterals can provoke CA stenosis by decreasing CA flow if it is already compromised by wall tension-increasing conditions such as median arcuate ligament syndrome or atherosclerosis [6]. In the presented case, the patient might have had CA stenosis before PDA aneurysm developed. However, according to the results of this study, it was equally possible that she already had the VAAs and collaterals due to other aortic branch compressions such as the iliac arteries during pregnancy [21]. After delivery, her PDA aneurysms might have remained and caused the CA stenosis to develop.

This study has some limitations. First, the present model simulated blood flow that is pulsatile with a direct current, which might affect the development of aneurysm [13, 17]. However, we do not believe that the flow pattern could have altered our conclusion since this study focused on the relationship between resistance and flow, which are independent of the flow's nature. Second, there could be a generalization problem that we chose CA stenosis among the various stenotic diseases of the major aortic branches and PDA aneurysm among the various VAAs [22, 23]. Although CA and PDA are two specific locations, no condition in this study requires specification of location of the stenosis or aneurysms, and we decided that the results of the current study could be applied without consideration of locations. Finally, the causal relationship between CA stenosis and PDA aneurysms, as shown in this study, is simply a possibility. More direct evidence is required to conclude which caused which, such as clinical evidence of the sequence of events or the recurrence of one condition after the correction of the other. Thus far, to our knowledge, there is only one case report in which a PDA aneurysm recurred at a distant site after transcatheter embolization of the ruptured PDA aneurysm with aortohepatic bypass for the concurrent CA stenosis [24]. This case suggests against the stenosis-first scenario. Despite these limitations, this study showed that the causal relationship between CA stenosis and PDA aneurysm could be bidirectional, contrary to common belief. There even exists a keen analogy between the arterial and portal venous sides of the splanchnic circulation in that the causal relationship between arterial stenosis and aneurysm (and collateral circulations) mimics liver cirrhosis and the portosystemic shunt [9]. We insist that the simulation of rare vascular diseases with electric equivalent circuits can be performed easily, which greatly helps our understanding as to what is going on and can predict the management results. This methodology should be used where the clinical study is difficult to perform and animal experiments are doomed to fail.

In conclusion, this study showed that in patients with concurrent CA stenosis and PDA aneurysm, either could come first and predispose the other. The simulation of splanchnic blood flow with an electric circuit provides a useful tool for analyzing rare vascular diseases that are difficult to provoke clinically or simulate in animal studies.

## Data Availability

The data used to support the findings of this study are included within the article.
